# Angiotensin-Converting Enzyme Inhibitors/Angiotensin Receptor Blockers: Anti-arrhythmic Drug for Arrhythmogenic Right Ventricular Cardiomyopathy

**DOI:** 10.3389/fcvm.2021.769138

**Published:** 2021-11-12

**Authors:** Bin Tu, Lingmin Wu, Lihui Zheng, Shangyu Liu, Lishui Sheng, Limin Liu, Zhenghui Zhu, Yan Yao

**Affiliations:** National Key Laboratory, Fuwai Hospital, National Center for Cardiovascular Diseases, Chinese Academy of Medical Sciences and Peking Union Medical College, Beijing, China

**Keywords:** angiotensin-converting enzyme inhibitors, arrhythmogenic right ventricular cardiomyopathy, anti-arrhythmic drug, ventricular arrhythmia, disease progression

## Abstract

**Background:** Current treatment guidelines for arrhythmogenic right ventricular cardiomyopathy (ARVC) mainly emphasize on prevention of ventricular arrhythmic events. Despite the progressive nature of ARVC, therapeutic options focusing on decelerating disease progression are scarce.

**Methods and Results:** This retrospective observational cohort study included 311 patients [age, 39.1 ± 14.4 years; male, 233 (74.9%)] with a definite diagnosis of ARVC as determined by the 2010 Task Force Diagnostic Criteria. Among them, 113 patients (36.3%) received ACEI/ARB treatment. Disease progression was evaluated according to repeat transthoracic echocardiograms with a linear mixed model. Patients receiving ACEI/ARB treatment were associated with slower disease progression reflected by a gradual decrease in tricuspid annular plane systolic excursion than those not receiving ACEI/ARB treatment (0.37 vs. 0.61 mm per year decrease, *P* < 0.001) and slower dilation of right ventricular outflow tract (0.57 vs. 1.06 mm per year increased, *P* = 0.003). Cox proportional hazard regression models were used to evaluate the association between life-threatening ventricular tachycardia events and ACEI/ARB treatment. A reduced risk of life-threatening ventricular arrhythmia was associated with ACEI/ARB treatment compared to that without ACEI/ARB treatment (adjusted HR: 0.71, 95% CI: 0.52–0.96, *P* = 0.031).

**Conclusions:** ACEI/ARB treatment is associated with slower disease progression and lower risk of life-threatening ventricular arrhythmia in patients with ARVC. Delaying disease progression may pave way for reducing life-threatening ventricular arrhythmia risk.

## Introduction

ARVC is a leading cause of sudden cardiac death/arrest (SCD/A) in young people and athletes (1). It is characterized by progressive replacement of the myocardium with fibrofatty tissue in the right ventricle (RV) and left ventricle (LV) in some cases (2). Continuous deterioration of the ventricles leads to ventricular arrhythmias (VA) and impaired ventricular function.

The primary goal of treatment is prevention of sudden cardiac death. Although implantable cardioverter defibrillators (ICDs) have been proven to be effective, repeated shock therapy, nevertheless, affects patients' quality of life (3). Attempts have been made to reduce the occurrence of VA by using classic anti-arrhythmic drugs and catheter ablation, but conflicting results have been reported (4–8). Some evidence suggests that the progressive nature of ARVC and persistent deterioration of the RV is associated with an increased risk of VA events (9–11). These findings indicate that therapies focused on delaying disease progression may offer a new approach for reducing the VA risk; however, no validated drug therapy has been established to decelerate disease progression.

Angiotensin-converting enzyme inhibitors/angiotensin receptor blockers (ACEI/ARB) are the standard treatment options for patients with systolic left ventricular dysfunction (12), and accumulating evidence points to their effectiveness in RV dysfunction (13–15). Additionally, ACEI/ARB treatment is related to a lower incidence of VA in patients with congestive heart failure (16). Therefore, in this study, we aimed to determine whether ACEI/ARB might slow disease progression in patients with ARVC and reduce the occurrence of VA events.

## Materials and Methods

### Study Population

Three hundred and fifty-four consecutive patients with a definite diagnosis of ARVC were enrolled, as determined by the revised 2010 Task Force Diagnostic Criteria (17), from October 2001 to November 2017 at a single tertiary national cardiac center in China. Patients who were lost to follow-up (*n* = 43) were excluded. In total, 311 patients were included for the final analysis. The baseline characteristic of patients with and without follow-up data were compared to confirm random loss; the results are shown in Supplementary Table S1. All medical records of the patients were systemically reviewed; written informed consent was obtained from all patients. The study was approved by the Institutional Ethics committee of Fuwai hospital.

### ACEI/ARB Medication

Medication status such as drug type, dosage, and compliance were recorded during routine follow-up, which was carried out every 6 months. For patients refilling prescriptions at our hospital, medication compliance was reconfirmed *via* the hospital's prescription web system. For follow-ups at patient homes, photographs of prescriptions were requested to confirm medication compliance. Patients were grouped based on ACEI/ARB use. Patients prescribed ACEI/ARB at some point during follow-up before the first episode of life-threatening VA (to those with recurrence of life-threatening VA) or until the end of follow-up (to those without recurrence of life-threatening VA) were included in the ACEI/ARB group. To increase the reliability of the investigation, sensitivity analyses with additional grouping strategy were conducted: (1) the duration of ACEI/ARB treatment exceeding 50% of the whole time until the first episode of life-threatening VA or until the end of follow-up; and (2) ACEI/ARB treatment continued until the first episode of life-threatening VA or until the end of follow-up. The results of sensitivity analyses are shown in Supplementary Table S2.

### Evaluation of Disease Progression

Disease progression was assessed by long-term surveillance of transthoracic echocardiograms (TTEs). To eliminate interobserver variability, TTE data were re-interpreted by a qualified observer blinded to the study and patient information. For intraobserver variability, TTE data of 50 random patients were re-interpreted by the same observer at least 1 month apart. The right ventricular outflow tract (RVOT) in the parasternal long-axis and tricuspid annular plane systolic excursion (TAPSE) were used to determine the right ventricular structure and function; left ventricular end-diastolic fraction and left ventricular ejection fraction (LVEF) were used to determine the left ventricular structure and function. All parameters were evaluated mainly based on two-dimensional echocardiography using standard processes as previously described (18).

### Follow-Up and Outcomes

Follow-ups were routinely carried out every 6 months *via* clinical visit or telephone consultation. The primary outcome was life-threatening VA events, including (1) SCD/A or aborted SCD/A; (2) sustained ventricular tachycardia (VT), lasting over 30 s and exceeding 100 bpm, with hemodynamic compromise or ventricular flutter/fibrillation; or (3) appropriate ICD shock. VA events were also detected *via* reports from a clinical network of initiatives consisting of dedicated patient phone lines, community primary care physician or specialist nurse reporting, and interval secondary/tertiary care outpatient reviews.

### Statistical Analyses

Statistical analyses were performed using R statistical software, version 3.4.3 (R Foundation for Statistical Computing, Vienna, Austria). Normally distributed continuous variables, non-normally distributed variables, and categorical variables are expressed as mean ± standard deviation (SD), median [interquartile range (IQR)], and number (percentage), respectively. Student's *t*-test, χ^2^ test, or the Mann-Whitney *U*-test was used for comparisons of baseline characteristics between the ACEI/ARB group and non-ACEI/ARB group. To maximize the statistical power of the study, we used a multivariate multiple imputation method to input missing data. Five imputed datasets were obtained, and a combined coefficient was calculated (see Supplementary Table S3). Sensitivity analyses were also performed to confirm missing randomized data. To evaluate disease progression, we used linear mixed models to analyze repeated measurements of TTE during follow-up. The model was adjusted for variables such as age sex, baseline values, and cardiac function. Cox proportional hazard regression models were used to analyze the association between life-threatening VA events and the use of ACEI/ARB. Model I was adjusted for confounders such as age, sex, RVEF, RV.LGE %, N-terminal pro-brain natriuretic peptide (NT-proBNP), ICD, and catheter ablation. Model II was additionally adjusted for LVEF, LV-LGE %, hypertension, and use of beta-blockers, spironolactone, and digoxin.

## Results

### Study Population Characteristics

Three hundred and eleven patients (average age: 39.1 ± 14.4 years; male: 233, 74.9%) with a definite diagnosis of ARVC were enrolled. The baseline characteristics are shown in Table 1. Of the 311 patients, 113 were prescribed ACEI/ARB after their first discharge from our hospital (ACEI: *n* = 68; ARB: *n* = 45) with a median treatment duration of 4.9 (IQR: 3.0–7.5) years from first clinical visit. Fifty-four patients had received ACEI/ARB treatment before they were first admitted to our hospital. Patients with hypertension and LV heart failure tended to be treated with ACEI/ARB, and hence, bi-ventricular dysfunction was more frequently observed in the ACEI/ARB group than in the non-ACEI/ARB group (*P* < 0.001). Sixteen patients discontinued ACEI/ARB usage during follow-up (10 for poor compliance, 4 for symptomatic hypotension at minimum dose, 1 for pregnancy, and 1 for end-stage renal dysfunction). The most prescribed ACEIs/ARBs were perindopril (*n* = 32) and losartan (*n* = 28). Four patients switched their ACEI medication to an ARB within 1 month because of cough, and they were considered a part of the ARB group for this analysis. Six patients started ACEI/ARB treatment with new onset symptomatic left ventricular dysfunction, and all of them had experienced the first episodes of life-threatening VA when the drug was taken, and all of them were considered a part of the non-ACEI/ARB group.

**Table 1:** Baseline clinical characteristics.

	**Overall**	**Without ACEI/ARB**	**ACEI/ARB**	***P*-value**
	**(*n* = 311)**	**(*n* = 198)**	**(*n* = 113)**	
Age (year)	39.1 ± 14.4	38.4 ± 14.8	40.3 ± 13.7	0.277
Male (%)	233 (74.9)	151 (76.3)	82 (72.6)	0.470
Body surface area (m^2^)	1.82 ± 0.2	1.80 ± 0.19	1.84 ± 0.22	0.150
Systolic blood pressure (mmHg)	115 ± 16	113 ± 15	117 ± 18	**0.032**
Heart rate (bpm)	69 ± 9	70 ± 8	69 ± 10	0.493
Syncope (%)	106 (34.1)	64 (32.3)	42 (37.2)	0.386
Cardiac function				** <0.001**
Biv dysfunction (%)	83 (27.5)	38 (19.2)	45 (39.8)	
Rv dysfunction (%)	148 (47.6)	108 (54.5)	40 (35.4)	
No dysfunction (%)	80 (25.7)	52 (26.3)	28 (24.8)	
Hypertension (%)	45 (14.5)	23 (11.6)	22 (19.5)	0.058
NT-proBNP, mmol/L (*n* = 275)	547 (245, 1,095)	567 (257, 979)	513 (232, 1,267)	0.932
**Electrocardiogram (*****n*** **=** **311)**				
TWI >3 (%)	133 (42.8)	88 (44.4)	45 (39.8)	0.428
Epsilon wave (%)	79 (26)	49 (24.8)	30 (28.3)	0.501
**24 h electrocardiogram monitoring (*****n*** **=** **205)**				
NSVT	93 (45.4)	58 (47.2)	35 (42.7)	0.529
24 h PVC count	1,349 (624, 4,351)	1,301 (624, 4,784)	1,504 (601, 3,332)	0.945
**Echocardiogram (*****n*** **=** **311)**				
LVED (mm)	48.6 ± 7.3	47.04 ± 6.21	51.42 ± 8.19	** <0.001**
LVEF (%)	60 (50, 65)	58.51 ± 10.91	52.20 ± 13.72	** <0.001**
RVOT (mm)	31.9 ± 9.7	31.2 ± 9.5	33.4 ± 10.0	0.057
TAPSE (mm)	16 (12,22)	16 (12,22)	16 (12,22)	0.998
**Cardiac magnetic resonance (*****n*** **=** **125)**				
LVEDV/body surface area	82.5 ± 35.2	75.85 ± 28.08	92.23 ± 41.95	**0.010**
LVEF	47.4 ± 14.4	50.99 ± 13.15	42.09 ± 14.61	** <0.001**
RVEDV/body surface area	148.2 ± 57.3	141.52 ± 51.20	157.42 ± 64.11	0.114
RVEF	25.2 ± 11.7	25.36 ± 10.93	24.92 ± 12.70	0.827
LV-LGE%	6.7 (1.97, 16.8)	4.2 (1.2, 11.3)	13.1 (4.3, 19.9)	**0.002**
RV-LGE%	16.9 (9.8, 28.0)	17.5 (10.1, 27.9)	15.1 (9.1, 27.8)	0.318
**Gene (*****n*** **=** **103)**				0.803
Single mutation (%)	69 (67.0)	18 (69.8)	51 (66.7)	
Compound mutation (%)	34 (33.0)	8 (30.2)	26 (33.3)	
**Electrophysiology study (*****n*** **=** **187)**				
Inducible VT/VF (%)	153 (81.8)	114 (84.4)	39 (75)	0.113
Late potential (%)	67 (35.8)	46 (34.1)	21 (40.4)	0.350
**Anti-arrhythmic drugs**				
Beta-blocker (%)	137 (44.1)	61 (30.8)	76 (67.3)	** <0.001**
Sotalol (%)	84 (27.0)	57 (28.8)	27 (23.9)	0.350
Amiodarone (%)	61 (19.6)	39 (19.7)	22 (19.5)	0.961
Spirolactone (%)	111 (35.6)	44 (22.2)	67 (59.3)	** <0.001**
Digoxin (%)	46 (14.8)	23 (11.6)	23 (20.4)	**0.037**
Implantable cardioverter defibrillator (%)	96 (30.9)	56 (28.3)	40 (35.4)	0.191
Ablation (%)	160 (51.4)	117 (59.1)	43 (38.1)	** <0.001**

*The bold values stand for P-value < 0.05; ACEI, angiotensin-converting enzyme inhibitors; ARB, angiotensin receptor blockers; Biv, bi-ventricular; Rv, Right ventricular; TWI, numbers of T wave inversion in pericardial lead; NSVT, non-sustained ventricular tachycardia; PVC, premature ventricular contractions; NT-proBNP, N-terminal pro-brain natriuretic peptide; LVED, left ventricular end-diastolic dimension; LVEF, left ventricular ejection fraction; RVOT, right ventricular outflow tract; TAPSE, tricuspid annular plane systolic excursion; LVEDV, left ventricular end-diastolic volume; RVEDV, right ventricular end-diastolic volume; RVEF, right ventricular ejection fraction; LV.LGE%, percentage of late gadolinium enhancement in the left ventricle; RV-LGE%, percentage of late gadolinium enhancement in right ventricle; VT/VF ventricular tachycardia/ventricular fibrillation*.

### Disease Progression

**Figure 1:**
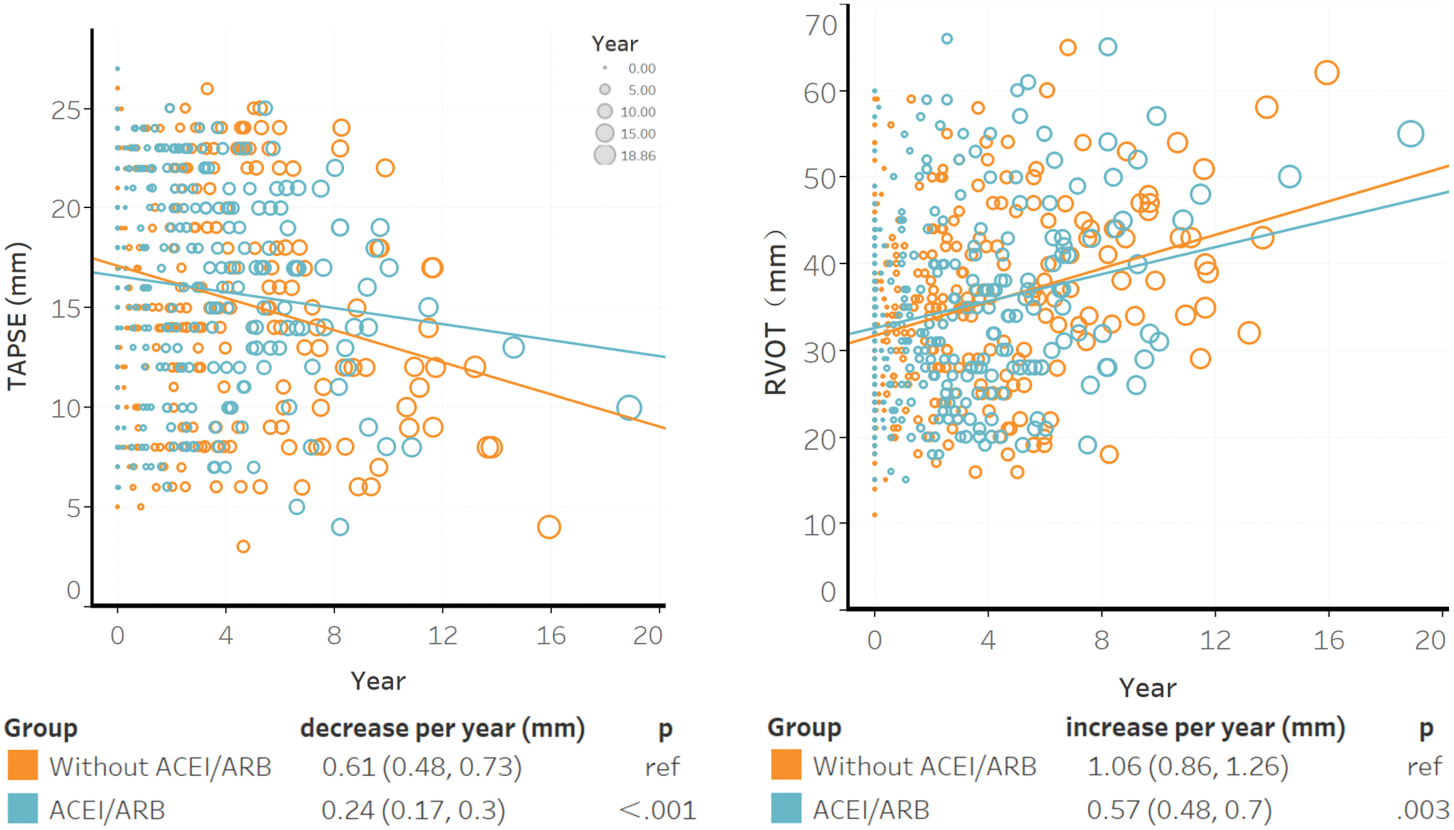
The association between ACEI/ARB treatment and right ventricular progression based on results of the transthoracic echocardiogram. Individual points were the unadjusted time to baseline TAPSE or RVOT during follow-up; larger circles represented longer time intervals from baseline. The blue trendline represents the progression speed in the ACEI/ARB group, and the orange trendline represents the progression in the non-ACEI/ARB group. Both trendlines were calculated using linear mixed models. ACEI, angiotensin-converting enzyme inhibitors; ARB, angiotensin receptor blockers; RVOT, right ventricular outflow tract; TAPSE, tricuspid annular plane systolic excursion.

Six hundred and thirty-seven TTEs were completed for 188 patients. According to the baseline characteristics shown in Supplementary Table S4, patients with recurrent life-threatening VA tended to undergo echocardiograms again. The median time between the baseline and the last TTE was 4.1 (IQR: 2.4–6.0) years. TAPSE tended to decline and RVOT tended to dilate over the years; however, ACEI/ARB decelerated this disease progression (Figure 1). In the non-ACEI/ARB group, TAPSE decreased by 0.61 mm per year [95% confidence interval (CI): 0.77–1.03]; this trend was slower in the ACEI/ARB group, in which TAPSE decreased to 0.24 mm per year (95% CI: 0.31–0.42), representing a 60.7% (95% CI: 58.9–64.5%, *P* < 0.001) relative reduction. Based on the test of power, 77.7% of patients might benefit from ACEI/ARB in terms of RV function restoration (α = 0.05; see Supplementary Table S4). Compared with ARB, ACEI elicited a strong effect and further delayed the declination of TAPSE by 70% (95% CI: 63.1–79.2%, *P* for interaction = 0.026). In patients with mild RV dysfunction, a slower progression was observed with ACEI/ARB treatment than without treatment (TAPSE: 0.37 vs. 0.61 mm per year decrease, *P* < 0.001; RVOT: 0.37 vs. 0.61 mm per year decrease, *P* < 0.001). For intraobserver variability, the ICC values were 0.91 (95% CI: 0.87–0.95) for RVOT-PLAX and 0.85 (95% CI: 0.76–0.94) for TAPSE.

### Life-Threatening VA Events

During the median follow-up of 5.96 (IQR: 3.74–8.61) years, 65.3% (*n* = 203) of the patients experienced life-threatening VA events, with an annual event rate of 19.8% (95% CI: 17.2–22.8%). The most common life-threatening VA events were sustained VT (*n* = 128), followed by ICD shock (*n* = 71) and SCD/A (*n* = 16). Twelve patients experienced various outcomes. In the multivariable analyses, relatively young age, male sex, a higher burden of 24-h premature ventricular contractions, recorded non-sustained VT, lower TAPSE, lower RVEF, and higher burden of RV-LGE% were associated with an increased risk of life-threatening VA in patients with ARVC (Table 2). The results of the univariate analyses are presented in Supplementary Table S6.

**Table 2:** Variables associated with life-threatening ventricular arrhythmia events.

	**Multivariable analysis**	
	**HR (95%CI)**	***P*-value**
**With TTE results**		
Age (per year decrease)	1.02 (1.01, 1.04)	0.007
Male	1.64 (1.04, 2.58)	0.032
NSVT	2.50 (1.52, 4.13)	<0.001
TWI ≥ 3	2.84 (1.96, 4.11)	<0.001
24 h PVC (In)	1.39 (1.23, 1.58)	<0.001
TAPSE (per mm decreased)	1.08 (1.05, 1.11)	<0.001
**With CMR results**		
24 h PVC count (In)	1.38 (1.12, 1.71)	0.003
TWI ≥ 3	2.47 (1.52, 4.03)	<0.001
RVEF (per % decreased)	1.03 (1.00, 1.05)	0.017
RV-LGE% (per % increased)	1.03 (1.01, 1.05)	0.001
ACEI/ARB	0.64 (0.39, 1.04)	0.072

*Hazard ratio (HR) and p-value were assessed using the Cox proportional hazard model. Variables without significant p-values in the multivariable analysis are not shown. NSVT, non-sustained ventricular tachycardia; TWI, numbers of T wave inversion in pericardial lead; PVC, premature ventricular complex; TAPSE, tricuspid annular plane systolic excursion; ACEI, angiotensin-converting enzyme inhibitors; ARB, angiotensin receptor blockers; RVEF, right ventricular ejection fraction; RV-LGE%, percentage of late gadolinium enhancement in right ventricle*.

### Association Between ACEI/ARB Medication and Recurrence of Life-Threatening VA

**Figure 2:**
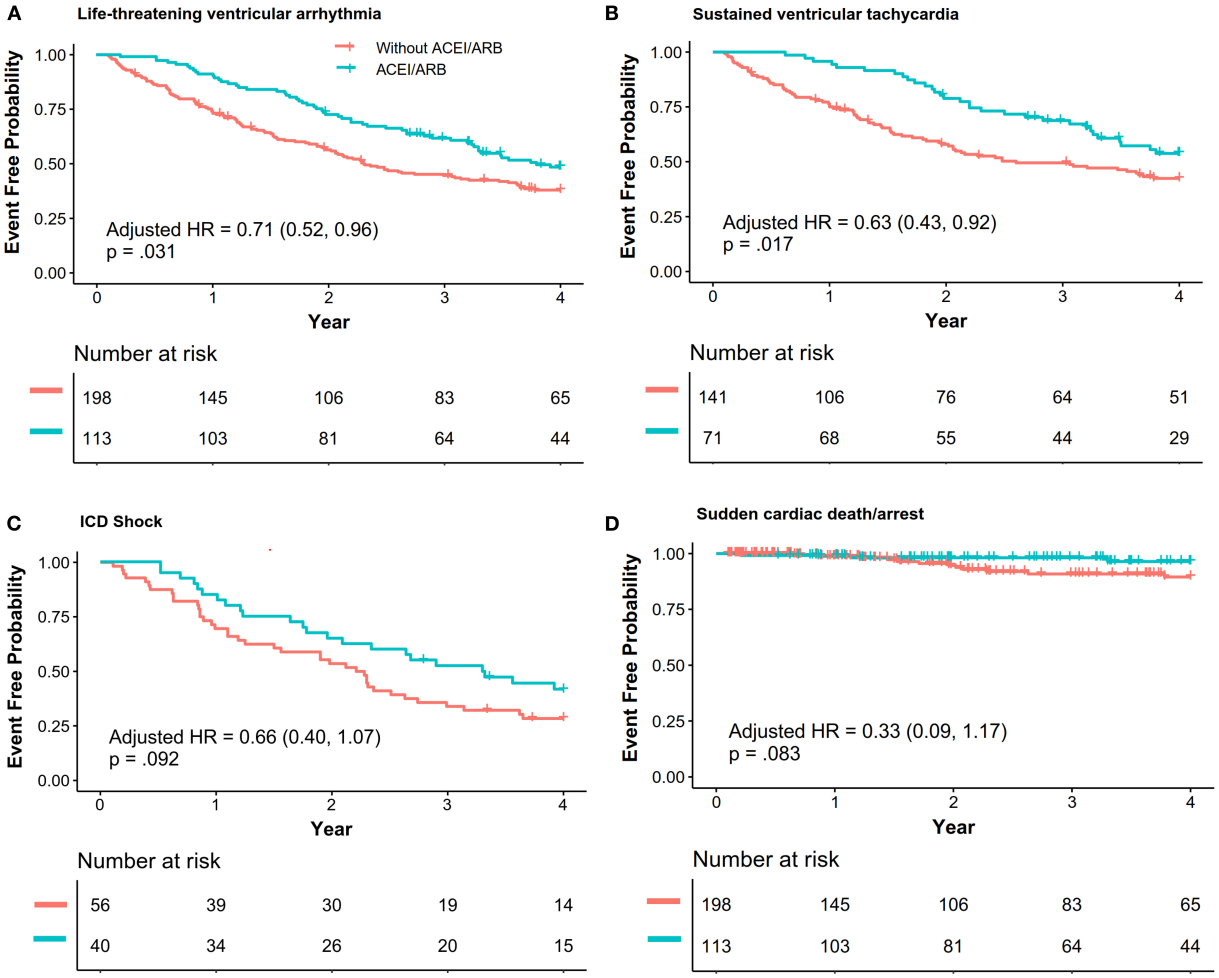
The association between ACEI/ARB treatment and recurrence of life-threatening ventricular VAs. Data were analyzed with composite outcomes as shown in **(A)**, and each outcome is individually shown in **(B–D)**. Hazard ratio (HR) and *p*-values were calculated *via* a Cox proportional hazard model, and the results were adjusted with Model I [right ventricular ejection fraction (baseline), percentage of late gadolinium enhancement in the right ventricle, N-terminal pro-brain natriuretic peptide, ICD, catheter ablation]. ACEI, angiotensin-converting enzyme inhibitors; ARB, angiotensin receptor blockers; VA, ventricular arrhythmia; VT, ventricular tachycardia; ICD, implanted cardiac defibrillator; SCD/A, sudden cardiac death/arrest.

**Figure 3:**
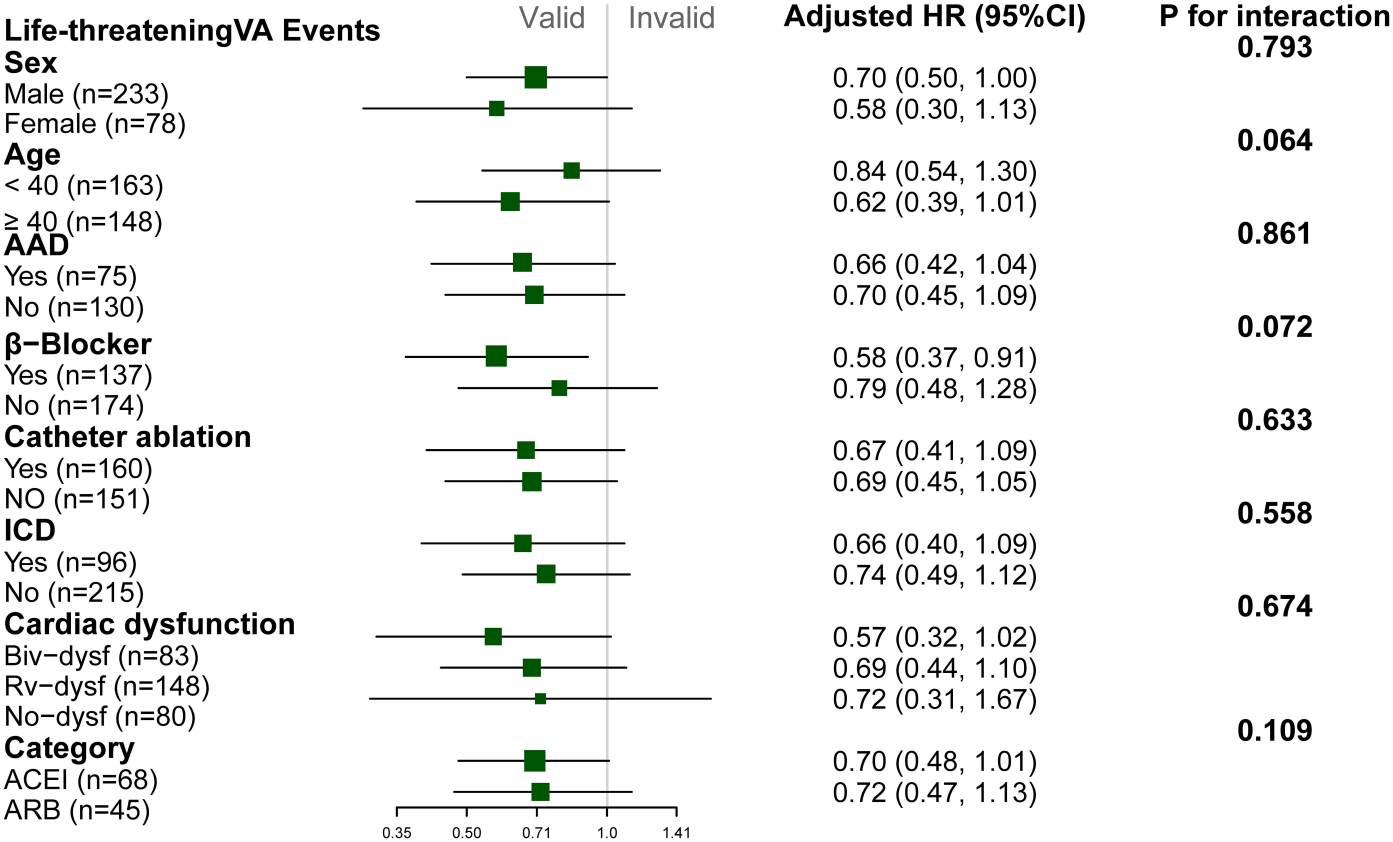
Subgroup analyses of the association between ACEI/ARB and recurrence of life threatening ventricular arrhythmia. Hazard ratio (HR) and *p*-values were calculated using a Cox proportional hazard model. All subgroup analyses were adjusted for Model I (right ventricular ejection fraction [baseline], N-terminal pro-brain natriuretic peptide, percentage of late gadolinium enhancement in right ventricle, catheter ablation, implanted cardiac defibrillator). No variable interacted with ACEI/ARB. NSVT, non-sustained ventricular tachycardia; PVC, premature ventricular complex; Biv-dysf, bi-ventricular dysfunction; Rv-dysf, right ventricular dysfunction; No-dysfunction, no ventricular dysfunction; ACEI, angiotensin-converting enzyme inhibitor; ARB, angiotensin receptor blocker.

Patients with ACEI/ARB had a lower occurrence of life-threatening VA than those not receiving ACEI/ARB [55.8 vs. 71.2%, crude hazard ratio (HR): 0.69, 95% CI: 0.51–0.93, *P* = 0.013]. This effect remained after adjustments in Model I (adjusted HR: 0.71, 95% CI: 0.52–0.96, *P* = 0.031) and Model II (adjusted HR: 0.68, 95% CI: 0.50–0.94, *P* = 0.018). Patients taking ACEI/ARB also showed a reduced risk of sustained VT (53.5 vs. 63.8%, adjusted HR: 0.63, 95% CI: 0.43–0.92, *P* = 0.017). Although HR favored a reduction in appropriated ICD shock (adjusted HR: 0.66, 95% CI: 0.40–1.07, *P* = 0.092) and SCD/A (2.7 vs. 6.6%, adjusted HR: 0.33, 95% CI: 0.09–1.17, *P* = 0.083), this did not reach statistical significance as shown in Figure 2. Results of the subgroup analyses are shown in Figure 3, with none of the variables showing significant association with ACEI/ARB.

## Discussion

To the best of our knowledge, this is the first single-center observational study to demonstrate the effects of ACEI/ARB in patients with ARVC during a long-term follow-up. The main findings were as follows. First, in addition to previously reported risk factors (9), a higher level of RV-LGE% was found to be associated with an increased risk of life-threatening VA in patients with ARVC. Second, ACEI/ARB treatment was associated with slower disease progression, which was reflected by a delay in the progress of RV dysfunction and dilation. Third, patients treated with ACEI/ARB had nearly one-third reduced risk of life-threatening VA compared to those without ACEI/ARB treatment.

The overall number of life-threatening VA events was higher in our study than those reported in previous studies (19). This might be due to the high-risk population enrolled in the present study; male sex, relatively young age, and extent of RV dysfunction facilitate arrhythmogenesis as determined both in previous and our studies (9, 10). The abnormal wall stretch caused by cardiac dysfunction may contribute to arrhythmogenesis through a process known as mechano-electrical feedback (20), which corroborates our findings that lower levels of TAPSE and RVEF and higher levels of NT-proBNP are associated with increased risk of life-threatening VA events. We found that higher RV-LGE% was also associated with an increased risk of life-threatening VA events, which correlated with the fact that fibrosis causes electric inhomogeneity and slow conduction, thereby facilitating arrhythmogenesis.

ACEI/ARB exhibit anti-remodeling effects in various types of cardiomyopathies (21, 22). This study revealed that ACEI also showed such an effect in patients with ARVC, which is compatible with the findings of Morel et al. (23), who showed that ACEI had an anti-fibrotic effect in patients with ARVC. Although the detailed pathogenesis of ARVC remains unclear, the major pathophysiological features include cardiomyocyte loss, fibrogenesis, and adipogenesis, which lead to the progression of ventricular dilation and dysfunction (24–26). According to the findings of previous studies, inhibition of apoptosis of cardiomyocytes through the P53 pathway and of fibrogenesis through the canonical and non-canonical transforming growth factor-β signaling pathways might explain this anti-remodeling (27, 28). ACEI showed a stronger anti-remodeling effect than ARB. Additional influence on the Kallikrein-Kinin/bradykinin system, angiotensin1–7, and ACE2 of ACEI likely contributed to this superior anti-remodeling effect (29–31).

Although ACEI/ARB are not classic anti-arrhythmic drugs, we found that patients with ARVC under ACEI/ARB treatment had a reduced risk of life-threatening VA events. This anti-arrhythmic effect has also been observed in other studies concerning left heart failure (16, 32). Additionally, ACEI also prevented newly onset or recurrence of atrial fibrillation due to substrate improvement (33). As mentioned above, inhibition of fibrogenesis might contribute to the anti-arrhythmic effect. Furthermore, reducing abnormal wall stretch of the ventricle by decreasing the preload and afterload of the RV might also lessen the risk of VA events (34–36). Stabilization of electrolyte concentration and prolonged action potential might also promote the anti-arrhythmic effect as suggested by other study (37).

ARVC is characterized by its arrhythmogenic property, but the underlying action is cardiomyopathy. Therefore, based on our study, anti-remodeling might be a potential therapeutic target for reducing VA risk; further randomized clinical studies are warranted to demonstrate this effect of ACEI/ARB.

This study has a few limitations. First, our study was limited by the inherent nature of retrospective cohort studies. Patients with hypertension and LV dysfunction tended to be treated with ACEI/ARB, and residual treatment bias inevitably affected our findings. Although we adjusted the LV dysfunction to generate more reliable results, another study showed that LV dysfunction also facilitated arrhythmogenesis (9), and indicated that patients on ACEI/ARB were at an increased risk for recurrence of life-threatening VA owing to a higher LV dysfunction, which emphasized the effectiveness of ACEI/ARB in reducing the risk of life-threatening VA. Second, imaging data for re-evaluation of the change in RV fraction area were available only up to 2010, when the revised Task Force Criteria were published; hence, this parameter was not considered, which resulted in the loss of a large amount of data. Third, RV size and systolic function were evaluated by two-dimensional echocardiography; even though there is some degree of association with three-dimensional results (18), there is also the potential for inaccuracy considering the complex geometry of the RV chamber.

## Conclusions

ARVC is a progressive disease characterized by continuous ventricular deterioration. A higher degree of RV dysfunction and fibrosis is associated with an increased risk of life-threatening VA. By reducing myocardial fibrosis and restoring RV function, ACEI/ARB might provide an anti-arrhythmic benefit to patients with ARVC.

## Data Availability Statement

The raw data supporting the conclusions of this article will be made available by the authors, without undue reservation.

## Ethics Statement

The studies involving human participants were reviewed and approved by Institutional Ethics Committee of Fuwai Hospital. Written informed consent to participate in this study was provided by the participants' legal guardian/next of kin.

## Author Contributions

YY conceived the study and formulated the analysis plan. LZ supervised the data collection. LL, SL, and LS collected and organized the clinical data and carried out the routine follow-up. LW performed the statistical analyses and edited the manuscript. ZZ re-interpreted the transthoracic echocardiogram results. BT wrote the manuscript. All authors contributed to the interpretation of the data, critical revision of the manuscript, and approval of the final version of the manuscript.

## Funding

This study was supported by the National Key R&D Program of China (Grant number 2017YFC1307800) and National Natural Science Foundation of China (Grant number 81800300). The funder had no role in the study design; in the collection, analysis, and interpretation of data; in the writing of the report; and in the decision to submit the article for publication.

## Conflict of Interest

The authors declare that the research was conducted in the absence of any commercial or financial relationships that could be construed as a potential conflict of interest.

## Publisher's Note

All claims expressed in this article are solely those of the authors and do not necessarily represent those of their affiliated organizations, or those of the publisher, the editors and the reviewers. Any product that may be evaluated in this article, or claim that may be made by its manufacturer, is not guaranteed or endorsed by the publisher.
