# Supplementary material for: Angiotensin-Converting Enzyme Inhibitors/Angiotensin Receptor Blockers: Anti-arrhythmic Drug for Arrhythmogenic Right Ventricular Cardiomyopathy
Source: Front Cardiovasc Med. 2021 Nov 12;8:769138. doi: 10.3389/fcvm.2021.769138 (PMC8632763; doi:10.3389/fcvm.2021.769138)
Supplement: Supplementary file 1 [file Data_Sheet_1.docx]

Supplementary Material

# Supplementary Tables

**Table S1**. **Observed characteristics comparison between participants with available and unavailable data**

311enrolled patient and 43 exclude patient without enough follow-up data. Only 24h PVC count show significant difference between group.

|  | **Enrollment**  **(n=311)** | **Exclude**  **(n=43)** | **p** |
| --- | --- | --- | --- |
| Age | 39.1±14.4 | 37.6±16.3 | 0.529 |
| Male | 233 (74.92) | 29 (67.44) | 0.295 |
| Probands | 283 (91) | 30 (93.75) | 0.600 |
| Hypertension | 45 (14.47) | 7 (16.28) | 0.753 |
| Body surface area | 1.81±0.20 | 1.78±0.16 | 0.415 |
| TWI＞3 | 133 (42.8) | 22 (51.16) | 0.172 |
| NSVT | 93 (45.37) | 9 (42.86) | 0.826 |
| 24h PVC count | 1349 (624, 4351) | 2634 (1501, 12887) | 0.014 |
| LVEF | 60 (50, 65) | 62 (56, 65) | 0.153 |
| RVEF | 32.76 (24.72, 44.20) | 36.38 (26.3, 44.4) | 0.908 |
| RVEDA | 10.68 (8.50, 12.99) | 10.60 (8.97, 13.35) | 0.638 |
| NT-proBNP | 547 (245, 1095) | 583 (413, 707) | 0.927 |
| cardiac function |  |  | 0.123 |
| No-dysf | 80 (25.72) | 14 (42.42) |  |
| Rv-dysf | 148 (47.59) | 12 (36.36) |  |
| Biv-dysf | 83 (26.69) | 7 (21.21) |  |
| Gene |  |  | 0.521 |
| Single mutation | 69 (67) | 4 (57) |  |
| Compound mutation | 34 (33) | 3 (43) |  |
| Ablation | 160 (51.45) | 20 (46.51) | 0.544 |
| ICD | 96 (30.87) | 10 (23.26) | 0.307 |

**Table S2. Sensitivity analyses of different grouping principal.**

|  | No. ACEI/ARB |  | **HR (95% CI)** | **P** |
| --- | --- | --- | --- | --- |
| Grouping principal 2 | n = 103 | Crude | 0.72 (0.54, 0.96) | 0.0081 |
|  |  | Model I adjustment | 0.70 (0.52, 0.95) | 0.0135 |
|  |  | Model II adjustment | 0.71 (0.51. 0.97) | 0.0289 |
|  |  |  |  |  |
| Grouping principal 3 | n = 97 | Crude | 0.69 (0.51, 0.93) | 0.0148 |
|  |  | Model I adjustment | 0.71 (0.52, 0.96) | 0.0282 |
|  |  | Model II adjustment | 0.72 (0.53, 0.98) | 0.0360 |

**Table S3. Missing Data input**

Missing data were inputting with 5 methods, and an overall HR was calculated.

| **Variable** | **Posterior predictive distribution** | **Predictive mean matching** | **Mean imputation** | **Median-imputation** | **Conditional mean-imputation** | **Overall** | **p** |
| --- | --- | --- | --- | --- | --- | --- | --- |
| Overall (crude) | 0.69  (0.51, 0.93) | 0.69  (0.51, 0.93) | 0.69  (0.51, 0.93) | 0.69  (0.51, 0.93) | 0.69  (0.51, 0.93) | 0.69  (0.51, 0.93) | 0.016 |
| Overall  (mode I) | 0.69  (0.51, 0.93) | 0.71  (0.53, 0.96) | 0.70  (0.52, 0.95) | 0.76  (0.56, 1.02) | 0.69  (0.51, 0.94) | 0.71  (0.52, 0.96) | 0.031 |
| Overall  (mode II) | 0.68  (0.50, 0.92) | 0.69  (0.51, 0.94) | 0.65  (0.48, 0.89) | 0.72  (0.53, 0.98) | 0.68  (0.50, 0.93) | 0.68  (0.50, 0.94) | 0.018 |
| Male  (n=233) | 0.69  (0.49, 0.97) | 0.71  (0.51, 1.00) | 0.69  (0.49, 0.98) | 0.75  (0.53, 1.06) | 0.70  (0.50, 0.99) | 0.70  (0.50, 1.00) | 0.052 |
| Female  (n=78) | 0.55  (0.29, 1.07) | 0.59  (0.31, 1.13) | 0.59  (0.30, 1.15) | 0.63  (0.33, 1.21) | 0.55  (0.29, 1.08) | 0.58  (0.30, 1.13) | 0.110 |
| Age＜40 (n=163) | 0.81  (0.54, 1.24) | 0.80  (0.52, 1.22) | 0.83  (0.54, 1.27) | 0.93  (0.61, 1.42) | 0.84  (0.55, 1.29) | 0.84  (0.54, 1.30) | 0.439 |
| ≥40  (n=148) | 0.61  (0.38, 0.97) | 0.65  (0.41, 1.03) | 0.61  (0.38, 0.98) | 0.66  (0.41, 1.07) | 0.62  (0.38, 1.00) | 0.62  (0.39, 1.01) | 0.058 |
| No-dysf (n=80) | 0.72  (0.31, 1.70) | 0.72  (0.31, 1.65) | 0.73  (0.32, 1.65) | 0.70  (0.30, 1.62) | 0.71  (0.30, 1.67) | 0.72  (0.31, 1.67) | 0.437 |
| Rv-dysf (n=148) | 0.66  (0.42, 1.03) | 0.73  (0.47, 1.14) | 0.70  (0.45, 1.09) | 0.74  (0.48, 1.16) | 0.64  (0.41, 1.00) | 0.69  (0.44, 1.10) | 0.119 |
| Bi-dysf  (n=83) | 0.55  (0.31, 0.97) | 0.58  (0.33, 1.01) | 0.54  (0.30, 0.96) | 0.62  (0.35, 1.09) | 0.60  (0.34, 1.04) | 0.57  (0.32, 1.02) | 0.063 |
| AAD  (n=139) | 0.67  (0.44, 1.02) | 0.70  (0.44, 1.09) | 0.64  (0.41, 1.00) | 0.70  (0.45, 1.09) | 0.63  (0.40, 0.97) | 0.66  (0.42, 1.04) | 0.079 |
| No AAD (n=172) | 0.66  (0.43, 1.03) | 0.68  (0.44, 1.03) | 0.72  (0.47, 1.09) | 0.77  (0.50, 1.17) | 0.68  (0.45, 1.04) | 0.70  (0.45, 1.09) | 0.115 |
| β-blocker  (n=137) | 0.54  (0.35, 0.82) | 0.57  (0.38, 0.87) | 0.53  (0.35, 0.80) | 0.66  (0.43, 1.00) | 0.60  (0.39, 0.91) | 0.58  (0.37, 0.91) | 0.018 |
| No β-blocker (n=174) | 0.78  (0.48, 1.25) | 0.82  (0.50, 1.33) | 0.79  (0.49, 1.28) | 0.80  (0.50, 1.30) | 0.75  (0.46, 1.22) | 0.79  (0.48, 1.28) | 0.337 |
| Ablation (n=160) | 0.65  (0.40, 1.04) | 0.70  (0.43, 1.12) | 0.66  (0.40, 1.08) | 0.69  (0.42, 1.11) | 0.65  (0.40, 1.04) | 0.67  (0.41, 1.09) | 0.107 |
| No abltion (n=151) | 0.71  (0.47, 1.06) | 0.69  (0.46, 1.04) | 0.65 (0.43, 0.99) | 0.74 (0.49, 1.12) | 0.67 (0.44, 1.02) | 0.69  (0.45, 1.05) | 0.088 |
| ICD  (n=96) | 0.64  (0.39, 1.06) | 0.65  (0.40, 1.05) | 0.65  (0.40, 1.08) | 0.71  (0.43, 1.16) | 0.67  (0.41, 1.09) | 0.66  (0.40, 1.09) | 0.108 |
| no-ICD (n=215) | 0.74  (0.49, 1.10) | 0.72  (0.48, 1.08) | 0.75  (0.50, 1.12) | 0.78  (0.52, 1.17) | 0.71  (0.47, 1.06) | 0.74  (0.49, 1.12) | 0.151 |
| AECI  (n=68) | 0.69  (0.48, 0.99) | 0.71  (0.49, 1.02) | 0.66  (0.46, 0.95) | 0.74  (0.52, 1.06) | 0.69  (0.48, 0.99) | 0.70  (0.48, 1.01) | 0.057 |
| ARB  (n=45) | 0.68  (0.44, 1.03) | 0.72  (0.47, 1.10) | 0.77  (0.50, 1.18) | 0.78  (0.51, 1.19) | 0.70  (0.46, 1.07) | 0.72  (0.47, 1.13) | 0.160 |

Biv-dysf, bi-ventricular dysfunction; Rv-dysf, right ventricular dysfunction; No-dysfunction, no ventricular dysfunction; AAD, anti-arrhythmia drug;

**Table S4. Observed characteristics comparison between with reperformed and without reperformed echocardiogram.**

|  | **Without reperformed echocardiogram**  (n=123) | **With reperformed echocardiogram**  (n=188) | **p** |
| --- | --- | --- | --- |
| Age | 40.2±14.9 | 38.3±13.9 | 0.241 |
| Male | 87 (70.16) | 146 (78.07) | 0.115 |
| Body surface area | 1.79±0.18 | 1.81±0.21 | 0.337 |
| Probands | 115 (92.74) | 168 (89.84) | 0.381 |
| Hypertension | 23 (18.6) | 22 (11.8) | 0.096 |
| Coronary heart disease | 8 (6.5) | 9 (4.8) | 0.534 |
| TWI＞3 | 49 (39.52) | 84 (44.92) | 0.346 |
| NSVT | 30 (35.71) | 63 (52.07) | 0.021 |
| 24h PVC count | 1403 (624, 3497) | 1346 (622, 4905) | 0.226 |
| LVEF | 60 (50, 65) | 60 (50, 65) | 0.656 |
| RVEF | 32.6 (20.7, 45.3) | 32.8 (25.5, 43.8) | 0.146 |
| RV-LGE% | 15.3 (8.1, 24.4) | 17.6 (10.1, 28.6) | 0.900 |
| NT-proBNP | 550 (204, 1032) | 539 (263, 1159) | 0.995 |
| cardiac function |  |  | 0.168 |
| No-dysfunction | 39 (31.45) | 41 (21.93) |  |
| Rv-dysfunction | 55 (44.35) | 93 (49.73) |  |
| Biv-dysfunction | 30 (24.19) | 53 (28.34) |  |
| ACEI/ARB | 34 (27.4) | 79 (42.3) | 0.008 |
| AAD | 51 (41.1) | 88 (47.1) | 0.303 |
| β-blocker | 49 (39.5) | 88 (47.1) | 0.190 |
| Ablation | 66 (53.2) | 94 (50.3) | 0.609 |
| ICD | 34 (27.4) | 62 (33.2) | 0.284 |
| Life threatening VA | 66 (53.23) | 137 (73.26) | <0.001 |

**Table S5.** The power (1-β) of test of ACEI/ARB function on TAPSE.

Carried out with repeating dotting (1000 times), and the results were listed below with each setting of α.

| **α** | **ACEI/ARB*Time** |
| --- | --- |
| <0.0001 | 162 |
| <0.001 | 324 |
| <0.01 | 580 |
| <0.05 | 777 |
| <0.1 | 852 |

**Table S6.** Univariable analysis of variable that associated with malignant ventricular arrhythmia recurrence and sensitivity analysis.

| **Univariable analysis** | | | | |
| --- | --- | --- | --- | --- |
|  | **Patients without ACEI/ARB treatment**  **(n= 198)** | | **All patients**  **(n=311)** | |
| Variable | HR (95%CI) | p | HR (95%CI) | p |
| Age (per year increased) | 0.99 (0.97, 1.00) | 0.024 | 0.98 (0.97, 0.99) | 0.001 |
| Male | 1.37 (0.89, 2.11) | 0.148 | 1.43 (1.02, 2.01) | 0.038 |
| Probands | 1.29 (0.68, 2.46) | 0.437 | 1.33 (0.80, 2.22) | 0.276 |
| Hypertension | 0.94 (0.55, 1.58) | 0.803 | 0.79 (0.53, 1.19) | 0.261 |
| NSVT | 3.33 (2.10, 5.27) | <0.001 | 3.56 (2.46, 5.14) | <0.001 |
| TWI≥3 | 2.60 (1.84, 3.67) | <0.001 | 2.50 (1.89, 3.32) | <0.001 |
| 24h PVC (In) | 1.47 (1.25, 1.73) | <0.001 | 1.41 (1.24, 1.60) | <0.001 |
| NT-proBNP (In) | 1.12 (0.97, 1.31) | 0.127 | 1.20 (1.06, 1.36) | 0.004 |
| Echocardiogram |  |  |  |  |
| LVED (per mm increased) | 1.00 (0.97, 1.03) | 0.948 | 0.99 (0.97, 1.01) | 0.354 |
| LVEF (per % increased) | 0.99 (0.97, 1.00) | 0.152 | 1.00 (0.98, 1.01) | 0.391 |
| RVOT (per mm increased) | 1.01 (0.99, 1.03) | 0.186 | 1.01 (1.00, 1.02) | 0.168 |
| TAPSE (per mm decreased) | 1.05 (1.02, 1.06) | <0.001 | 1.06 (1.03, 1.08) | <0.001 |
| Cardiac magnetic resonance |  |  |  |  |
| LVEDVi (per mm^3^ increased) | 1.00 (0.99, 1.01) | 0.762 | 1.00 (1.00, 1.01) | 0.293 |
| LVEF (per % decreased) | 1.01 (0.99, 1.04) | 0.196 | 0.99 (0.97, 1.00) | 0.131 |
| RVEDVi (per mm^3^ increased) | 1.00 (1.00, 1.01) | 0.141 | 1.00 (1.00, 1.01) | 0.0539 |
| RVEF (per % decreased) | 1.05 (1.02, 1.07) | <0.001 | 1.05 (1.03, 1.07) | <0.001 |
| LV-LGE% (per % increased) | 1.01 (0.98, 1.04) | 0.708 | 1.00 (0.98, 1.02) | 0.875 |
| RV-LGE% (per % increased) | 1.04 (1.02, 1.06) | <0.001 | 1.04 (1.03, 1.06) | <0.001 |
| EP study |  |  |  |  |
| Inducible VTs | 1.35 (0.75, 2.43) | 0.314 | 1.18 (0.74, 1.88) | 0.496 |
| Late potential | 0.94 (0.62, 1.43) | 0.768 | 0.95 (0.66, 1.37) | 0.801 |
| Fragmented potential | 1.22 (0.71, 2.10) | 0.466 | 1.23 (0.74, 2.06) | 0.425 |
| Treatment |  |  |  |  |
| Beta blocker | 1.23 (0.86, 1.75) | 0.251 | 1.01 (0.77, 1.34) | 0.922 |
| ACEI/ARB |  |  | 0.69 (0.51, 0.93) | 0.013 |
| [Spirolactone](javascript:;) | 0.98 (0.65, 1.49) | 0.933 | 0.94 (0.71, 1.26) | 0.691 |
| Digoxin | 0.96 (0.58, 1.60) | 0.881 | 1.33 (0.93, 1.91) | 0.123 |
| Sotalol | 1.26 (0.88, 1.81) | 0.208 | 1.21 (0.89, 1.63) | 0.225 |
| [Amiodarone](javascript:;) | 1.42 (0.95, 2.12) | 0.092 | 1.38 (1.00, 1.93) | 0.054 |
| [Catheter](javascript:;) [ablation](javascript:;) | 1.04 (0.72, 1.51) | 0.829 | 1.08 (0.81, 1.43) | 0.610 |
| ICD | 1.42 (0.99, 2.04) | 0.057 | 1.34 (1.00, 1.79) | 0.047 |

HR and P values were carried out with Cox regression model. In multivariable analysis variable without statistically significance were excluded. RVEF, right ventricular ejection fraction; NSVT, non-sustained ventricular tachycardia; PVC, premature ventricular complex; TWI, numbers of T wave inversion in pericardial lead; NT-proBNP, N-terminal pro-brain natriuretic peptide; RV.LGE%, percentage of late gadolinium enhancement in right ventricle.
